# A population genomics appraisal suggests independent dispersals for bitter and sweet manioc in Brazilian Amazonia

**DOI:** 10.1111/eva.12873

**Published:** 2019-10-19

**Authors:** Alessandro Alves‐Pereira, Charles R. Clement, Doriane Picanço‐Rodrigues, Elizabeth Ann Veasey, Gabriel Dequigiovanni, Santiago Linorio Ferreyra Ramos, José Baldin Pinheiro, Anete Pereira de Souza, Maria Imaculada Zucchi

**Affiliations:** ^1^ Departamento de Genética Escola Superior de Agricultura “Luiz de Queiróz” Universidade de São Paulo (ESALQ‐USP) Piracicaba Brazil; ^2^ Departamento de Biologia Vegetal Instituto de Biologia Universidade Estadual de Campinas (UNICAMP) Campinas Brazil; ^3^ Instituto Nacional de Pesquisas da Amazônia (INPA) Manaus Brazil; ^4^ Instituto de Ciências Biológicas Universidade Federal do Amazonas (UFAM) Manaus Brazil; ^5^ Agência Paulista de Tecnologia dos Agronegócios Pólo Centro‐Sul (APTA) Piracicaba Brazil

**Keywords:** Amazonian crops, domestication, genomics, *Manihot esculenta*, RADseq, single nucleotide polymorphism

## Abstract

Amazonia is a major world centre of plant domestication, but the genetics of domestication remains unclear for most Amazonian crops. Manioc (*Manihot esculenta*) is the most important staple food crop that originated in this region. Although manioc is relatively well‐studied, little is known about the diversification of bitter and sweet landraces and how they were dispersed across Amazonia. We evaluated single nucleotide polymorphisms (SNPs) in wild and cultivated manioc to identify outlier SNPs putatively under selection and to assess the neutral genetic structure of landraces to make inferences about the evolution of the crop in Amazonia. Some outlier SNPs were in putative manioc genes possibly related to plant architecture, transcriptional regulation and responses to stress. The neutral SNPs revealed contrasting genetic structuring for bitter and sweet landraces. The outlier SNPs may be signatures of the genomic changes resulting from domestication, while the neutral genetic structure suggests independent dispersals for sweet and bitter manioc, possibly related to the earlier domestication and diversification of the former. Our results highlight the role of ancient peoples and current smallholders in the management and conservation of manioc genetic diversity, including putative genes and specific genetic resources with adaptive potential in the context of climate change.

## 1 INTRODUCTION

Amazonia is widely recognized as an area of great interest to humanity due to its immense biological and cultural diversity (Heckenberger, Russell, Toney, & Schmidt, 2007; Laurance et al., 2001). Amazonia is also a major world centre of plant domestication (Clement, 1999a; Meyer, DuVal, & Jensen, 2012), which should not be surprising given such biocultural diversity. Plant domestication is a coevolutionary process in which humans select useful plants that become better adapted to domesticated landscapes (Clement, 1999a). Pre‐Columbian peoples domesticated at least 83 native crops, most of them in the periphery of the Amazon basin (Clement, 1999a; Clement, de Cristo‐Araújo, D’Eeckenbrugge, Alves Pereira, & Picanço‐Rodrigues, 2010). Some Amazonian crops are of current global importance, such as manioc (*Manihot esculenta* Crantz), cacao (*Theobroma cacao* L.) and peanuts (*Arachis hypogaea* L.). Among these, manioc (also known as cassava) is a major staple food crop cultivated around the Tropics and is the main source of calories for about 800 million people (Lebot, 2009). Moreover, manioc cultivation will be of increasing importance for food security during climate change (Burns, Gleadow, Cliff, Zacarias, & Cavagnaro, 2010; Howeler, Lutaladio, & Thomas, 2013).

Cultivated manioc (*M. esculenta* ssp. *esculenta*) was domesticated from *M. esculenta* ssp. *flabellifolia* (Pohl) Cif. (Allem, 1994; Léotard et al., 2009; Olsen, 2004; Olsen & Schaal, 1999, 2001). Although some controversy persists, these studies support a single domestication in the south‐western Amazon basin. This is because the genetic variability of cultivated manioc is a subset of that found in ssp. *flabellifolia* populations occurring in the Brazilian states of Rondônia, Mato Grosso and Acre, and northern Bolivia (Léotard et al., 2009; Olsen, 2004; Olsen & Schaal, 2001). *Manihot esculenta* ssp. *flabellifolia* occurs in forest/savannah ecotones growing as a climbing vine in open forests and as a highly branched bush in savannahs (Ménard, McKey, Mühlen, Clair, & Rowe, 2013). Cultivated manioc was selected for increased tuberous root yields and for vegetative propagation (McKey & Delêtre, 2017). The selection for clonal reproduction resulted in less‐branched plants with thicker and more brittle stems than the wild relative (Elias, Lenoir, & McKey, 2007; Ménard et al., 2013).

Divergent selective pressures originated the two major groups of domesticated landraces: sweet manioc and bitter manioc (McKey, Cavagnaro, Cliff, & Gleadow, 2010). Sweet manioc has a lower content of toxic cyanogenic glycosides (CNglcs) (<100 ppm fresh weight) than bitter manioc (>100 ppm fresh weight), which demands more careful detoxification (McKey et al., 2010). Although the CNglc content varies continuously among bitter and sweet landraces, these groups are genetically divergent and the farmers distinguish them (Elias, Mühlen, McKey, Roa, & Tohme, 2004; Mühlen, Martins, & Ando, 2000). Interestingly, cultivated manioc still reproduces sexually, which can lead to gene flow among distinct landraces and hybridization with wild *Manihot* (McKey, Elias, Pujol, & Duputié, 2012). The sexual seeds remain in soil seed banks and may sprout in newly opened swiddens (Duputié, Massol, David, Haxaire, & McKey, 2009; Martins, 2007; Pujol, Renoux, Elias, Rival, & McKey, 2007), where farmers may keep these volunteer seedlings until harvest (Elias, Rival, & McKey, 2000; Rival & McKey, 2008). When farmers use sexual plants for clonal propagation, they may either incorporate them into an existing landrace or create a new one (Duputié et al., 2009; Martins, 2007). The traditional management of manioc is also characterized by exchange networks of landraces within and among traditional communities (Boster, 1986; Chernela, 1986), promoting the diffusion of landraces over large geographic areas (Delêtre, McKey, & Hodkinson, 2011). Sexual reproduction and the incorporation of volunteer seedlings, plus the exchange networks, contribute to create and conserve the great genetic diversity of manioc.

Apparently, manioc was dispersed quickly in the Neotropics. Considering an initial domestication about 10,000 years before present (BP) (Olsen & Schaal, 1999), archaeobotanical data suggest the occurrence of manioc along the Peruvian Pacific coast by 8,000 BP and an ample Neotropical occurrence by 6,500 BP (Isendahl, 2011). Using linguistic evidence, Brown, Clement, Epps, Luedeling, and Wichmann (2013) suggested that manioc became important in Amazonia before 4,000 BP, when sedentary horticultural societies started to thrive in Amazonia. However, we do not know if other wild *Manihot* spp. were used before domesticated manioc landraces, and thus, these dates may not precisely reflect the distribution of cultivated manioc (Brown et al., 2013). Moreover, much less is known about the genetics of the crop's dispersal in Amazonia than about its origins, although the current distribution of bitter and sweet manioc cultivation may provide clues (Emperaire, 2001; McKey & Beckerman, 1993). While bitter manioc cultivation is prevalent along the major Amazonian rivers, and along the eastern coasts of South America, sweet manioc cultivation is prevalent in the headwaters of the major rivers and on a smaller scale where bitter manioc dominates. Emperaire (2001) attributed these contrasting distributions to a limited history of interchange of bitter and sweet landraces among human populations, which possibly reflects independent dispersals of these groups of landraces.

The order of the creation of bitter and sweet manioc is still a subject of speculation. McKey and Beckerman (1993) review different hypotheses involving either the initial selection of sweet or bitter manioc from a sweet or bitter wild ancestor, respectively; the independent selection of bitter and sweet manioc from respective bitter and sweet wild ancestors; and simultaneous selection of bitter and sweet manioc from the same wild ancestor. Additionally, Arroyo‐Kalin (2010) hypothesized that sweet manioc was first domesticated by small‐scale forager‐incipient horticulturalist populations. Bitter manioc would have been selected only around 4,000–3,000 BP, when food production was intensified in Amazonia and technology for detoxification was fully developed (Arroyo‐Kalin, 2010). Because Perrut‐Lima, Mühlen, and Carvalho (2014) classified extant populations of ssp. *flabellifolia* in the centre of manioc's domestication as bitter, another hypothesis emerged based on their findings and Arroyo‐Kalin’s (2010) ideas. Sweet manioc was initially selected from ssp. *flabellifolia* populations, possibly of intermediate to high toxicity, and bitter manioc was later selected from sweet landraces. Some genetic studies appear to support this latter hypothesis (Alves‐Pereira et al., 2018; Mühlen, Alves‐Pereira, Clement, & Valle, 2013), but they did not discard alternative scenarios.

In the last two decades, our knowledge about plant domestication has been greatly enhanced by molecular genetic studies (Larson et al., 2014). In this period, the emergence of next‐generation sequencing technologies (NGS) enabled the generation of large numbers of DNA sequences (Metzker, 2010). NGS eventually became a powerful tool for the discovery and assessment of genetic markers (Davey et al., 2011) and fostered the use of single nucleotide polymorphisms (SNPs) in the emerging field of population genomics (Black, Baer, Antolin, & DuTeau, 2001; Siol, Wright, & Barrett, 2010). Generally, thousands of SNPs are discovered and it is possible to separate those that follow neutral expectations from outlier markers putatively under selection (Luikart, England, Tallmon, Jordan, & Taberlet, 2003). Such approaches are attractive because the outlier SNPs may be applied for the study of adaptation while genome‐wide neutral SNPs generally improve the resolution of population genetic studies (Luikart et al., 2003). Indeed, population genomics significantly improved our knowledge about plant domestication (Gepts, 2014; Kantar, Nashoba, Anderson, Blackman, & Rieseberg, 2017; Morrell, Buckler, & Ross‐Ibarra, 2012).

Much effort has been made to generate genomic information for manioc since the release of a genome for the crop (Prochnik et al., 2012). Some NGS‐based studies evaluated the genome‐wide diversity of the crop and wild relatives, but they were primarily applied to the crop's genetic improvement (Albuquerque et al., 2019; ICGMC, 2015; Pootakham et al., 2014). Additionally, they used few Amazonian landraces, and little attention was given to the evolution of the crop in this region (Bredeson et al., 2016; Ramu et al., 2017). Information about genetic variation within the centre of domestication is essential to conserve the crop's diversity and its potential to adapt to climate change (Howeler et al., 2013). In this context, we identified genome‐wide SNP markers in bitter, sweet and wild manioc to evaluate putative signs of selection and the patterns of genetic diversity along the major rivers in Brazilian Amazonia. With this population genomics approach, we continue the evolutionary analysis of bitter and sweet manioc under traditional cultivation in Amazonia started by our group with chloroplast and nuclear microsatellite markers (Alves‐Pereira et al., 2018). We chose this approach to better characterize the putative adaptive variation and improve the resolution of the analyses performed previously with neutral markers. Based on our previous results, we focused on two aspects of the evolution of manioc under traditional management. (a) The signs of selection putatively related to the domestication and diversification of the crop. We identified considerable genetic divergence between wild and cultivated manioc, and between bitter and sweet manioc landraces with microsatellite (SSR) markers (Alves‐Pereira et al., 2018). Therefore, we expect to provide new information about possible signatures of genomic changes resulting from the selection of manioc landraces based on the identification of SNP markers putatively under selection. (b) What the structuring of genome‐wide diversity of wild and cultivated manioc may suggest regarding the dispersal of the crop in Amazonia. We expect that genome‐wide neutral SNP markers will provide a robust estimation of the relationships among the groups of bitter, sweet and wild manioc, as well as illuminate patterns of their genetic structure. With this information, we will continue to evaluate unanswered questions from our previous study (Alves‐Pereira et al., 2018), including the genetic evidence for the order of creation of the cultivated landraces and the possible dispersal routes of bitter and sweet manioc in Amazonia.

## 2 MATERIALS AND METHODS

### 2.1 Sampling sites

**Figure 1:**
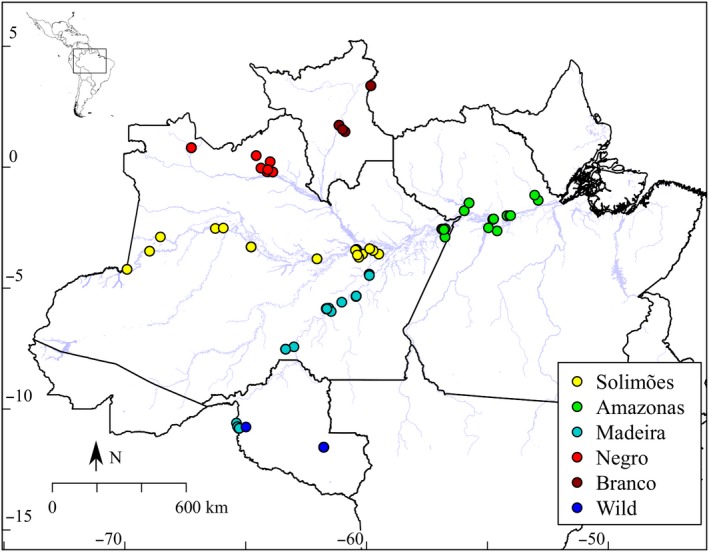
Geographic locations of wild and cultivated manioc (*Manihot esculenta* Crantz) sampled in communities of smallholder farmers along the major Amazonian rivers (Negro, Branco, Madeira, Solimões and Amazonas) in Brazil

For the identification of SNP markers, we used a subset of 159 landraces sampled previously (Alves‐Pereira et al., 2018). We included in this sample 71 bitter manioc, 69 sweet manioc and 19 *M. esculenta* ssp. *flabellifolia* (hereafter wild manioc). We sampled one wild manioc near a field of cultivated manioc and 18 individuals in the surroundings of the experimental station of the Federal University of Rondônia (UNIR), Rolim de Moura (Table S1), where Perrut‐Lima et al. (2014) also collected. Bitter and sweet landraces are from communities of smallholder farmers in 24 municipalities along the Negro, Branco, Madeira, Solimões and Amazonas rivers (Figure 1; Table S1). In each community, we explained the study's objectives, and sampling was exclusively of leaves, which were dehydrated with silica gel and posteriorly maintained at −20°C. We collected one leaf of only one plant for each landrace cultivated in the swiddens or homegardens. We also obtained photographs and geographic locations of swiddens/homegardens (recorded with a GPS). We registered our research (number A7994B4) in Brazil's Council for Genetic Patrimony (CGEN), according to Law 13,123 (20 May 2015).

Our population genomic analyses were primarily based on the groupings of manioc samples by their reputed toxicity and domestication status (i.e. bitter, sweet and wild manioc). We recognize that these groups of bitter, sweet and wild manioc are not “true populations.” However, along with clonal propagation, sexual reproduction still occurs in manioc (McKey & Delêtre, 2017), most (if not all) traditional landraces are polyclonal (Martins, 2007), and social networks promote the exchange of landraces over a wide geographic scale (Delêtre et al., 2011). Therefore, in this study we treated these groups of landraces as rough approximations of metapopulations of manioc traditionally grown in Brazilian Amazonia.

### 2.2 DNA isolation, preparation and sequencing of the genomic library

We obtained genomic DNA from 50 mg of manioc leaf tissue using either DNeasy Plant Mini Kit (Quiagen) or NucleoSpin Plant II (Macherey‐Nagel) following the manufacturers' instructions. We inspected the quality and quantified DNA samples with electrophoresis in agarose 1% (w/v) gels stained with SYBR safe DNA (Invitrogen) by comparison with Phage lambda molecular size standards (Invitrogen). After quantification, we normalized DNA samples to 30 ng/µl.

Library preparation using the restriction site‐associated DNA (RAD) technique (Baird et al., 2008) and sequencing were performed by Floragenex Inc. (USA). Briefly, ~300 ng of genomic DNA was digested with the endonuclease *Sbf*I, and the resulting fragments were ligated to adapters containing unique barcodes (proprietary of Floragenex Inc.) for each sample. Individual samples were then pooled and randomly sheared, and DNA fragments ranging from 300 to 800 bp were size‐selected in 1.5% (w/v) agarose gel and recovered with a MinElute Gel Extraction Kit (Qiagen). An adapter complementary to the Illumina's flow cell was ligated to the fragments, and the library was enriched for RAD fragments through PCR. To increase sequencing throughput, RAD libraries were prepared for two sets of manioc samples, with 87 and 72 landraces, and each library was sequenced in two lanes of an Illumina HiSeq 2,500 (Illumina), with a single‐end (100‐bp) configuration.

### 2.3 Identification of SNP markers

Proprietary bioinformatic scripts were used by Floragenex Inc. to obtain FASTQ files and to demultiplex samples according to their unique barcodes. We aligned the resulting 91‐bp reads against the manioc genome *Manihot esculenta v6.1* (Prochnik et al., 2012) using *Bowtie v.2.2.1* (Langmead & Salzberg, 2012). We performed alignments involving all bases of a read using the “end‐to‐end” and “sensitive” configurations, allowing up to one mismatch (*‐N 1*) between each read and the reference genome. SNP discovery was performed using *SAMtools v.0.1.19* (Li, 2011; Li et al., 2009) and *VCFtools v.0.1.13* (Danecek et al., 2011). We retained only one SNP per RAD tag to avoid explicit linkage between markers. In addition, we considered only the SNPs with a minimum depth of 5X, minor allele frequency ≥0.01, mapping quality ≥13 and present in at least 90% of the samples of each bitter, sweet and wild manioc. Sequence alignments (bam files) were deposited in Sequence Read Archive of the National Center for Biotechnology Information (SRA‐NCBI, accession PRJNA532929). Table S1 contains the SNP data used for the analyses (in variant call format).

### 2.4 Detection and characterization of SNPs putatively under selection

A number of tests for the identification of loci putatively under selection (outliers) have been developed (Lotterhos & Whitlock, 2014, 2015). However, because they may not accurately account for population structure and its covariance with other variables, such as demography and mutation rates, false positives may be present (Lotterhos & Whitlock, 2014, 2015; Narum & Hess, 2011). A general attempt to counteract this issue is the application of different tests (Jordan, Hoffmann, Dillon, & Prober, 2017). Therefore, we performed three analyses (*fsthet*, *BayeScan* and *pcadapt*) to take advantage of their different models.

*Fsthet* (Flanagan & Jones, 2017) and *BayeScan* (Foll & Gaggiotti, 2008) are methods for the detection of outlier loci based on Wright’s (1943) fixation index *F*_ST_. *Fsthet* is similar to the *fdist2* method (Beaumont & Nichols, 1996), which identifies loci with excessively high or low *F*_ST_ (under positive and balancing selection, respectively) compared to the expected *F*_ST_‐*H_E_* relationships in an island model of migration (Wright, 1931). Because the *F*_ST_‐*H*_E_ distribution may change depending on genetic structure, *fsthet* uses smoothed quantiles based on the empirical distribution of *F*_ST_‐*H*_E_ to identify outliers, without assuming a particular model of evolution (Flanagan & Jones, 2017). In contrast, *BayeScan* decomposes locus‐population *F*_ST_ into beta (population‐specific) and alpha (locus‐specific) components using a logistic regression (Foll & Gaggiotti, 2008). A positive value of alpha suggests diversifying selection, whereas negative values suggest balancing or purifying selection. For each locus, *BayeScan* estimates the posterior probability of including or not including the alpha component to model selection using reversible‐jump Markov chain Monte Carlo (RJ‐MCMC), considering the island model of migration and the uncertainty of allele frequencies (Foll & Gaggiotti, 2008). Unlike the methods above, *pcadapt* does not require pre‐defined populations and does not assume any genetic model (Luu, Bazin, & Blum, 2017). *Pcadapt* evaluates the genetic structure through principal component analysis (PCA) and generates a vector of *z*‐scores, which measure the relationships of each SNP marker to the first *K* principal components. Then, a Mahalanobis distance (Maronna & Zamar, 2002) is computed for each SNP to detect outliers for which the vector of *z*‐scores does not follow the distribution of the majority of points (Luu et al., 2017).

While *pcadapt* requires no groupings, we used the a priori groupings of bitter, sweet and wild manioc in *fsthet* and *BayeScan*. We used *fsthet 1.0.1* (Flanagan & Jones, 2017) for R (R Core Team, 2018) to calculate the *F*_ST_ and *H*_E_ based on the variance in allele frequencies corrected for sample sizes with the estimator $\hat{\beta }$ (Cockerham & Weir, 1993). The smoothed confidence intervals were generated with 1,000 bootstrap replicates, and candidate outliers were identified as the most extreme in the two sides of distribution, considering alpha = 0.05. Using *BayeScan 2.1* (Foll & Gaggiotti, 2008), we generated 20 pilot runs, each with 5,000 iterations of the RJ‐MCMC, followed by a burn‐in of 50,000 plus 150,000 RJ‐MCMC iterations, with a sample size of 5,000 and a thinning interval of 20. We identified candidate outliers based on q‐values ≤ 0.05 (analogue to FDR: 5% of the outliers are expected to be false positives). We used *pcadapt 4.03* (Luu et al., 2017) for R (R Core Team, 2018) to select the number of *K* principal components (with *K* varying from 1 to 20) to perform the test. We chose *K* = 3 because the addition of more components did not increase significantly the proportion of the variance explained by PCA (Figure S1), and considered as outliers the loci with *q*‐values ≤0.10 (Luu et al., 2017). We considered candidate outliers as the loci that deviated from neutral expectations under at least two of these three tests, and distinguished loci putatively under positive or balancing selection according to the results of *fsthet* and BayeScan. Additionally, because *pcadapt* is based on PCA, we were able to infer whether the outlier SNPs were related to the genetic divergence between wild and cultivated manioc (first principal component) or to the divergence between bitter and sweet landraces (second principal component; Figure S1).

We obtained the predicted effects of outlier SNPs using *SnpEff* (Cingolani et al., 2012). Functional characterization based on the annotations for the *Manihot esculenta v6.1* gene models (i.e. predicted genes) with outlier SNPs was performed using the online tools of *Phytozome v12* (http://www.phytozome.jgi.doe.gov/pz/portal.html). We used *PhytoMine* (Kalderimis et al., 2014; Smith et al., 2012) to recover Gene Ontology (GO) term annotations, which summarize the information about cellular components, molecular functions and biological processes (Blake, 2013) putatively related to predicted genes with outlier SNPs. Additionally, we used BLASTN (Basic Local Alignment Search Tool of Nucleotides), with default configurations, to search for similarities between the predicted genes in manioc and those of *Arabidopsis thaliana* deposited in The Arabidopsis Information Resource (TAIR10) (Lamesch et al., 2012). We evaluated the most significant hit for each BLASTN and recorded the putative or well‐described protein coded by the respective *Arabidopsis* gene in UniProt (http://www.uniprot.org).

### 2.5 Genomic diversity analyses

For these analyses, we considered only the putatively neutral SNPs. We estimated the number of multilocus genotypes (*MLGs*), total number of alleles (*A*), number of private alleles (*Ap*), observed (*H*_O_) and expected (*H*_E_) heterozygosities and Wright's (1965) inbreeding coefficient (*f*). We used *diveRsity* (Keenan, McGinnity, Cross, Crozier, & Prodöhl, 2013), *poppr* (Kamvar, Tabima, & Grünwald, 2014) and *PopGenKit* (Paquette, 2012) for R (R Core Team, 2018) to obtain these estimates and confidence intervals for *H*_O_, *H*_E_ and *f*, based upon 1,000 bootstrap replicates. We evaluated the distribution of genetic variation within and among groups of manioc landraces with analyses of molecular variance (AMOVA) in *Arlequin 3.5* (Excoffier & Lischer, 2010) and tested their significance with 20,000 permutations.

We evaluated genetic structure by estimating pairwise Weir and Cockerham’s (1984) *F*_ST_ among bitter, sweet and wild manioc, and among cultivated landraces from different rivers, and their significances based upon 1,000 bootstraps, with *hierfstat* (Goudet & Jombart, 2015). Additionally, we performed discriminant analysis of principal components (DAPC) (Jombart, Devillard, & Balloux, 2010) with *adegenet* (Jombart & Ahmed, 2011) for R (R Core Team, 2018). DAPC is versatile because it does not rely on a particular population genetics model (Jombart et al., 2010). We performed DAPCs based on groupings of bitter, sweet and wild manioc, and groupings of cultivated manioc by rivers, to investigate the genetic relationships and the admixture among landraces.

We evaluated the clustering of landraces and rivers with neighbour‐joining (Saitou & Nei, 1987) dendrograms built with *Phylip 3.7* (Felsenstein, 2009), based on Cavalli‐Sforza and Edwards’ (1967) Chord distance (*D*_CE_) obtained with *MSA 4.05* (Dieringer & Schlötterer, 2003). *D*_CE_ is a Euclidian distance and estimates well the relationships among related individuals (Reif, Melchinger, & Frisch, 2005). We assessed confidence of clustering with 1,000 bootstrap replicates and formatted the consensus trees in *FigTree 1.4.1* (http://www.tree.bio.ed.ac.uk/software/figtree/).

## 3 RESULTS

### 3.1 Identification of SNPs putatively under selection

Sequencing of RAD libraries resulted in a total of 401,551,249 reads, with a mean of 2,525,479.6 (*SD* ± 1,350,765.5) reads per individual. Our restrictive filtering resulted in a final set of 2,013 SNP markers, with no missing data, a mean depth of 109.8 (*SD* ± 5.2) per individual and a mean per‐site SNP quality of 955 (*SD* ± 196). From these, we detected 216 candidate outliers with *fsthet*, 195 with *pcadapt* and only three with *BayeScan*. Larger *q*‐values (>0.1) increased the number of candidate outliers detected with *BayeScan* but resulted in less statistical power. Because all these three candidate outliers were also detected with *pcadapt*, we opted to maintain this very conservative result as evidence of selection. In total, 46 SNPs were considered outliers by at least two of these tests: 44 putatively under positive selection and two putatively under balancing selection (Tables S2 and S3). Based on *pcadapt*, we found that 43 SNPs putatively under positive selection were related to wild versus cultivated divergence (principal component 1 in Figure S1b). Additionally, the two SNPs putatively under balancing selection were related to the bitter–sweet divergence (principal component 2 in Figure S1b).

**Figure 2:**
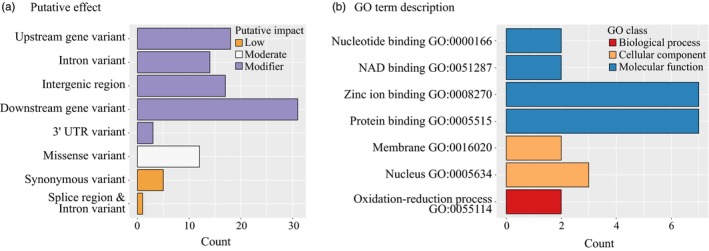
Annotation of SNP markers putatively under selection performed for 159 bitter, sweet and wild manioc (*Manihot esculenta*). (a) Bar plot of the predicted effects of 46 outlier SNPs on their associated genomic regions. (b) Bar plot of the most common functional annotations of the transcripts with SNP markers putatively under selection, summarized as Gene Ontology classification (GO terms). Table S4 contains the complete list of predicted effects and GO terms

Because most of the outlier SNPs were within either introns or non‐coding regions, the predicted effects of these variants were mostly “Modifier”, that is mutations not involved in amino acid change. On the other hand, most of the predicted effects in exons were missense mutations (Figure 2a; Table S3). We found that 29 candidate SNP outliers were in *Manihot esculenta v6.1* predicted genes, of which 15 were in exons, 12 in introns and two in 3′‐untranslated regions. Among the 29 manioc predicted genes with an outlier SNP, 21 had at least one associated GO term. We found a great variety of GO terms (55), but only seven of them occurred in more than one predicted gene (Figure 2b). Although most GO terms were unique, many of them referred to binding to inorganic or organic molecules, enzymatic activity, metabolism and transcription (Table S3). Additionally, 27 manioc predicted genes had sequence similarity with genes of *Arabidopsis thaliana*. We focus our discussion on a subset of 12 of these genes, which are putatively involved in root architecture, plant growth and development, responses to biotic and abiotic stresses and transcriptional regulation (Table 1). In the discussion below, we speculate how selective pressures on this subset of genes may have been important for the domestication and diversification of manioc. The complete list of the predicted genomic effects of outlier SNPs in manioc predicted genes, and their GO annotations and similarity with *Arabidopsis* genes, is in Table S3.

**Table 1:** Predicted effects and annotation of 12 SNP markers putatively under selection and their associated *Manihot esculenta v6.1* predicted genes for the groups of bitter, sweet and wild manioc (*Manihot esculenta*) grown along major Amazonian rivers in Brazil

ID^a^	SNP^b^	Manioc predicted gene	Predicted effect^c^	GO terms	Similar to	Putative function in *Arabidopsis*
SNP_320 (p1)	G > A	Manes.03G047100	Intron variant (Modifier)	None	AT5G56750.1: N‐MYC downregulated‐like 1 (NDL1)	NDL1 acts regulating lateral root formation (Mudgil et al., 2009) and regulating meristem initiation and branching (Mudgil, Ghawana, & Jones, 2013)
SNP_1787 (p1)	T > A	Manes.16G103800	Missense variant (Moderate): [239]Arg > Ser	None	AT5G56750.1: N‐MYC downregulated‐like 1 (NDL1)	Idem.
SNP_135 (p1)	G > A	Manes.01G221600	Missense Variant (Moderate): [520]Pro > Leu	GO:0004857 (enzyme inhibitor activity), GO:0005618 (cell wall), GO:0030599 (pectinesterase activity), GO:0042545 (cell wall modification)	AT1G02810.1: Plant invertase/pectin methylesterase inhibitor superfamily (PME7)	PME7 contributes to both firming and softening of cell walls (Louvet et al., 2006)
SNP_672 (b2)	A > G	Manes.06G003200	Intron variant (Modifier)	GO:0000166 (nucleotide binding), GO:0003700 (transcription factor activity, sequence‐specific DNA binding), GO:0005515 (protein binding), GO:0005634 (nucleus), GO:0006355 (regulation of transcription, DNA‐templated), GO:0008270 (zinc ion binding), GO:0015079 (potassium ion transmembrane transporter activity), GO:0016020 (membrane), GO:0071805 (potassium ion transmembrane transport)	AT1G10170.1: ATNFXL1, NFXL1	NFXL1 encodes a transcription factor required for growth under salt stress and likely involved in protection of photosynthesis (Lisso, Altmann, & Müssig, 2006). NFXL1 may act in the response to phytopathogenic fungi, and general defence response in *Arabidopsis* (Asano et al., 2008)
SNP_1282 (p1)	C > T	Manes.11G134800	3′UTR variant (Modifier)	GO:0005515 (protein binding), GO:0008270 (zinc ion binding)	AT5G10380.1: RING/U‐box superfamily protein (ATRING1, RING1, ALT55)	ATL55 may be involved in general plant defence signalling pathways (Lin et al., 2008)
SNP_377 (p1)	T > G	Manes.03G128100	Synonymous variant (Low): [39]Pro > Pro	None	AT5G25610.1: BURP domain‐containing protein (ATRD22, RD22)	RD22 acts in drought stress response, suppressing chlorophyll degradation under moisture stress (Harshavardhan et al., 2014)
SNP_456 (p1)	C > G	Manes.04G024600	Intron variant (Modifier)	GO:0005634 (nucleus), GO:0008270 (zinc ion binding), GO:0016049 (cell growth), GO:0016925 (protein sumoylation), GO:0019789 (SUMO transferase activity), GO:0031668 (cellular response to extracellular stimulus)	AT5G60410.2: DNA‐binding protein with MIZ/SP‐RING zinc finger, PHD‐finger and SAP domain (ATSIZ1, SIZ1)	SIZ1 participates in the regulation of phosphate deficiency responses (Miura et al., 2005), freezing tolerance (Miura et al., 2007), drought tolerance (Catala et al., 2007) and facilitates basal thermotolerance (Yoo et al., 2006). It is involved in the regulation of plant growth and flowering (Catala et al., 2007; Jin et al., 2008)
SNP_601 (p1)	T > G	Manes.05G114400	Missense Variant (Moderate): [260]Ser > Ala	GO:0003677 (DNA binding)	AT1G25340.1: MYB domain protein 116 (AtMYB116, MYB116)	MYB116 has a DNA‐binding MYB domain. MYB proteins are a superfamily of transcription factors involved in regulation of developmental processes and defence responses in plants (Yanhui et al., 2006)
SNP_301 (p1)	A > G	Manes.03G023700	3′UTR variant (Modifier)	GO:0046983 (protein dimerization activity)	AT4G29100.1: basic helix‐loop‐helix (bHLH) DNA‐binding superfamily protein (BHLH68)	BHLH68 probably encodes a transcription factor (Riechmann et al., 2000) with a bHLH DNA‐binding domain (Bailey et al., 2003)
SNP_355 (p1)	T > C	Manes.03G095300	Intron variant (Modifier)	GO:0006357 (regulation of transcription from RNA polymerase II promoter), GO:0032777 (Piccolo NuA4 histone acetyltransferase complex), GO:0035267 (NuA4 histone acetyltransferase complex)	AT1G79020.1: Enhancer of polycomb‐like transcription factor protein (YUP8H12R.36)	YUP8H12R.36 probably encodes a transcription factor (Riechmann et al., 2000) involved in many developmental transitions, including vernalization (Chanvivattana et al., 2004; Derkacheva & Hennig, 2014)
SNP_1648 (p1)	G > A	Manes.15G081800	Missense Variant (Moderate): [562]Asp > Asn	GO:0005515 (protein binding), GO:0008270 (zinc ion binding), GO:0016579 (protein deubiquitination), GO:0036459 (ubiquitinyl hydrolase activity)	AT3G20630.1: ubiquitin‐specific protease 14 (ATUBP14, PER1, TTN6, UBP14)	UBP14 is involved in seed and embryo development (Doelling, Yan, Kurepa, Walker, & Vierstra, 2001; Tzafrir et al., 2002)
SNP_85 (p1)	G > T	Manes.01G131000	Intron variant (Modifier)	None	AT1G06660.1: JASON	JASON is required for normal spindle orientation at male meiosis II and normal formation of tetrad of microspores (De Storme & Geelen, 2011)

^a^ ID = SNP identification (p = positive selection, b = balancing selection, 1 = wild‐cultivated divergence, 2 = bitter–sweet divergence).

^b^ SNP = *Manihot esculenta v6.1* reference versus alternative allele.

^c^ Predicted effect = change predicted within the primary transcript of the associated manioc predicted gene. When the predicted effect is an amino acid substitution, it is coded as [number of the residue] original amino acid > new amino acid.

### 3.2 Patterns of genomic diversity and genetic structure

We evaluated how the genomic diversity was organized across bitter, sweet and wild manioc based on 1,985 neutral SNPs. These markers had great discrimination power, since the number of multilocus genotypes (*MLGs*) was equal to the sample number for all groupings of manioc landraces. Bitter and sweet manioc had similar numbers of alleles, but bitter manioc had almost twice as many private alleles as sweet manioc (Table 2). Bitter manioc had a significantly greater deficit of heterozygotes than sweet manioc (*f* = 0.122 and 0.048, respectively; see non‐overlapping confidence intervals in Table 2). Wild manioc had more private alleles (191) than either bitter or sweet manioc, but fewer private alleles than the cultivated set of manioc landraces (964). Although wild manioc had significantly lower estimates of genetic diversity than bitter and sweet manioc, it had the lowest deficit of heterozygotes (Table 2). Estimates of genetic diversity were similar across bitter and sweet landraces from different rivers, but bitter manioc from different rivers had greater deficits of heterozygotes than the groups of sweet manioc (Table 2).

**Table 2:** Estimates of genomic diversity and inbreeding based on 1,985 neutral SNP markers for wild and cultivated manioc (*Manihot esculenta*), and the major Amazonian rivers in Brazil

Manioc	*N*	*A*	*A*_P_	*H*_O_ (95% CI)	*H*_E_ (95% CI)	*f* (95% CI)
Bitter	71	3,678	112	0.169 (0.161, 0.177)	0.192 (0.185, 0.200)	0.122* (0.106, 0.139)
Sweet	69	3,605	58	0.169 (0.160, 0.179)	0.178 (0.170, 0.186)	0.048* (0.007, 0.085)
Cultivated	140	3,779	964	0.169 (0.161, 0.177)	0.195 (0.188, 0.202)	0.131* (0.114, 0.147)
Wild	19	3,006	191	0.149 (0.139, 0.159)	0.157 (0.148, 0.166)	0.047 (−0.045, 0.128)
Rivers						
Madeira bitter	19	3,376	5	0.169 (0.160, 0.177)	0.186 (0.178, 0.193)	0.091* (0.048, 0.124)
Negro bitter	16	3,270	11	0.165 (0.156, 0.175)	0.176 (0.168, 0.184)	0.062* (0.018, 0.096)
Branco bitter	7	3,043	2	0.168 (0.158, 0.178)	0.170 (0.162, 0.178)	0.014 (−0.114, 0.086)
Solimões bitter	17	3,344	6	0.172 (0.163, 0.182)	0.183 (0.176, 0.191)	0.060* (0.016, 0.093)
Amazonas bitter	12	3,298	3	0.170 (0.162, 0.180)	0.186 (0.179, 0.195)	0.085* (0.003, 0.141)
Madeira sweet	18	3,250	5	0.167 (0.157, 0.177)	0.167 (0.159, 0.175)	0.001 (−0.071, 0.058)
Negro sweet	9	2,832	0	0.177 (0.165, 0.190)	0.143 (0.136, 0.152)	−0.233* (−0.487, −0.084)
Branco sweet	8	3,198	6	0.166 (0.157, 0.175)	0.183 (0.176, 0.191)	0.096 (−0.022, 0.159)
Solimões sweet	16	3,151	2	0.174 (0.164, 0.185)	0.167 (0.160, 0.175)	−0.042 (−0.126, 0.034)
Amazonas sweet	18	3,340	28	0.166 (0.157, 0.175)	0.181 (0.174, 0.189)	0.086* (0.006, 0.145)

Abbreviation: *N*, number of individuals.

Genetic diversity: *A* = number of alleles, *A*_P_ = number of private alleles, *H*_O_ = observed heterozygosity, *H*_E_ = expected heterozygosity, *f* = Wright's inbreeding coefficient, (95% CI) = 95% confidence interval.

* *f* significantly different from zero based upon 1,000 bootstrap replicates.

AMOVAs suggested that most of the genetic variation was within groups (Table 3). The highest divergence was between wild and cultivated manioc (*Φ*_ST_ = 0.286), followed by the divergence between bitter and sweet manioc (*Φ*_ST_ = 0.086). Although we observed significant divergence among rivers (*Φ*_ST_ = 0.071), most of this was due to genetic differences between bitter and sweet manioc within rivers (*Φ*_SC_ = 0.107). Based on pair‐wise *F*_ST_, sweet manioc was a little more divergent from wild manioc (*F*_ST_ = 0.321) than bitter manioc (*F*_ST_ = 0.297), but these estimates were not significantly different from each other (Table 4). When considering bitter and sweet landraces grouped by rivers, most divergences were low to intermediate (*F*_ST_ < 0.1), with higher divergences restricted to comparisons between bitter and sweet landraces from different rivers (Table 4). Overall, genetic divergences among sweet manioc from different rivers (average pairwise *F*_ST_ = 0.027) were greater than divergences among bitter manioc from different rivers (average pairwise *F*_ST_ = 0.003).

**Table 3:** Analysis of molecular variance (AMOVA) based on 1,985 neutral SNP markers for different groups of wild and cultivated manioc (*Manihot esculenta*) grown along major Amazonian rivers in Brazil

Source of variation	*df*	Sums of squares	Variance components	Percentage of variation	*Φ* statistics
Between wild and cultivated (*N* = 159)	1	5,286.85	76.17	28.63	*Φ*_ST_ = 0.286*
Within wild and cultivated	316	59,999.88	189.87	71.37	
Between bitter and sweet^a^ (*N* = 140)	1	2,635.98	17.51	8.64	*Φ*_ST_ = 0.086*
Within bitter and sweet	278	51 ,460.89	185.11	91.36	
Among rivers^a^ (*N* = 140)	4	1,439.99	7.86	−4.04	*Φ*_CT_ = −0.040
Between bitter and sweet within rivers	5	3,873.08	21.72	11.17	*Φ*_SC_ = 0.107*
Within rivers	270	48,782.93	180.68	92.88	*Φ*_ST_ = 0.071*

Abbreviations: *df*, degrees of freedom; *N*, sample size in each hierarchical level.

* Significant at *p* < .001.

^a^ Disregarding wild manioc.

**Table 4:** Pairwise *F*_ST_ (Weir & Cockerham, 1984) estimates based on 1,985 neutral SNP markers for different groups of wild and cultivated manioc (*Manihot esculenta*) grown along major Amazonian rivers in Brazil

Manioc	Bitter	Sweet	Cultivated						
Bitter	–								
Sweet	0.086	–							
Wild	0.297	0.321	0.285						
Rivers	MB	NB	BB	SB	AB	MS	NS	BS	SS
Madeira bitter (MB)	–								
Negro bitter (NB)	0.045	–							
Branco bitter (BB)	0.038	0.039	–						
Solimões bitter (SB)	0.030	0.019	0.021	–					
Amazonas bitter (AB)	0.026	0.044	0.038	0.034	–				
Madeira sweet (MS)	0.068	0.133	0.122	0.114	0.081	–			
Negro sweet (NS)	0.116	0.192	0.194	0.169	0.140	0.019	–		
Branco sweet (BS)	0.052	0.098	0.075	0.078	0.043	0.025	0.079	–	
Solimões sweet (SS)	0.092	0.152	0.145	0.131	0.097	0.004	0.036	0.021	–
Amazonas sweet (AS)	0.061	0.113	0.098	0.097	0.060	0.017	0.050	0.003	0.015

Note

*F*_ST_ between AS and BS was the only non‐significant estimate based upon 1,000 bootstrap replicates.

**Figure 3:**
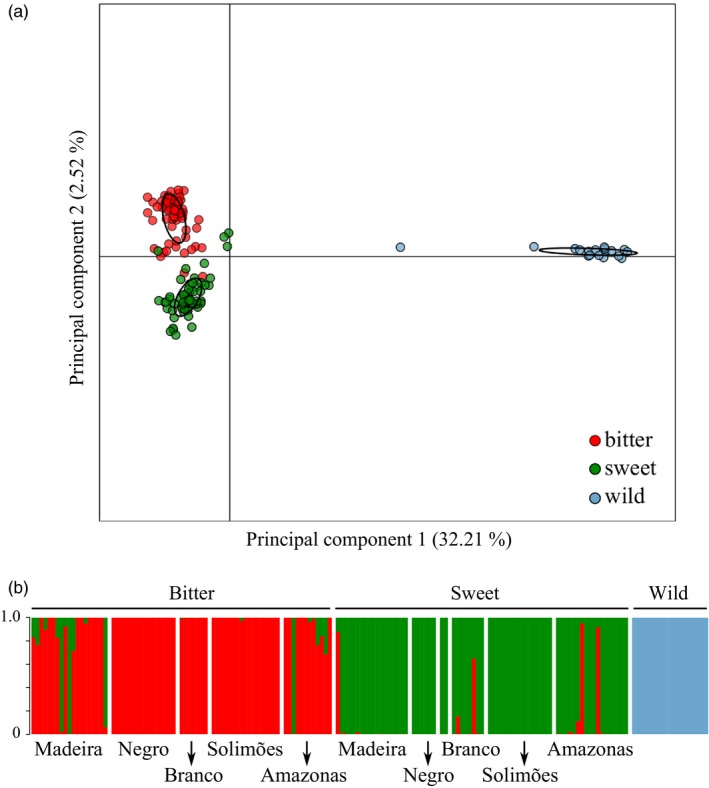
Discriminant analysis of principal components (DAPC) based on 1,985 neutral SNP markers for 159 cultivated landraces and wild manioc individuals (*Manihot esculenta*) from the major Amazonian rivers in Brazil. (a) Scatterplot illustrating the dispersion, with the respective standard deviation around the centroid (black ellipses), of bitter, sweet and wild manioc. (b) Bar plot of DAPC membership coefficients, with bitter and sweet landraces sorted by rivers

In the DAPC performed for the whole set of individuals, wild manioc formed a very distinct cluster from the cultivated landraces, while bitter and sweet manioc presented some overlap (Figure 3a). Considering an arbitrary threshold of 80% for the membership coefficients, five cultivated landraces were admixed (four reputed bitter and one reputed sweet). Additionally, three reputed sweet landraces clustered within the group of bitter manioc, and four reputed bitter landraces clustered within the group of sweet manioc (Figure 3b; Table S4).

**Figure 4:**
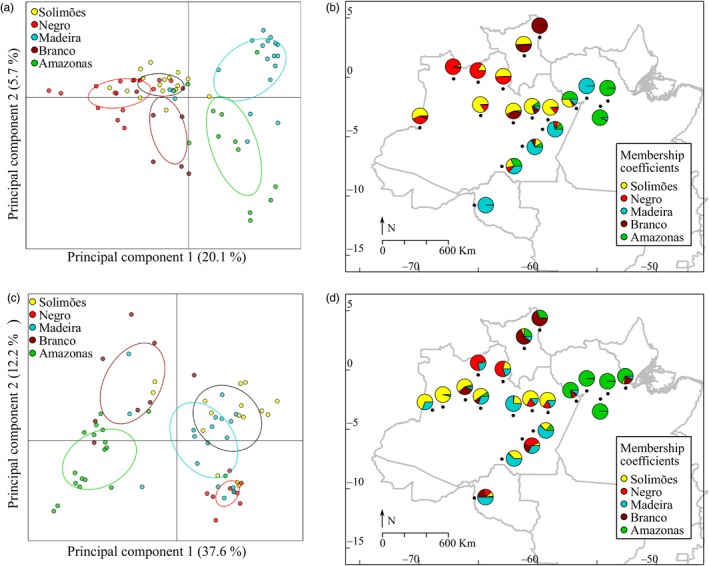
Genetic structure of bitter and sweet manioc (*Manihot esculenta*) landraces from the major Amazonian rivers in Brazil based on 1,985 neutral SNP markers. DAPC scatterplots illustrating the dispersion of (a) 71 bitter and (c) 69 sweet manioc landraces, with the respective standard deviation around the centroid (ellipses). Maps showing the distribution of DAPC membership coefficients averaged over the (b) bitter and (d) sweet manioc collected in the different municipalities. The colours are the same used in scatterplots, and circle sizes are not proportional to sampling sizes

In general, the DAPCs performed separately for bitter and sweet manioc showed considerable admixture for the groups of landraces from different rivers (Figure 4; Table S4). The bitter and sweet landraces from the Madeira River and the bitter landraces from the Solimões River showed considerable dispersion across the other groups. For bitter manioc, the first two components explained 25.8% of total genetic variation. Bitter landraces from the Madeira and Amazonas Rivers tended to cluster closer to each other, while landraces from the Solimões, Negro and Branco Rivers were more dispersed, with considerable overlap (Figure 4a,b). For sweet manioc, the first two components explained 49.7% of total genetic variation. Sweet landraces from the Branco and Amazonas Rivers clustered closer to each other, while landraces from the Madeira, Solimões and Negro Rivers were more dispersed, with considerable overlap (Figure 4c,d).

**Figure 5:**
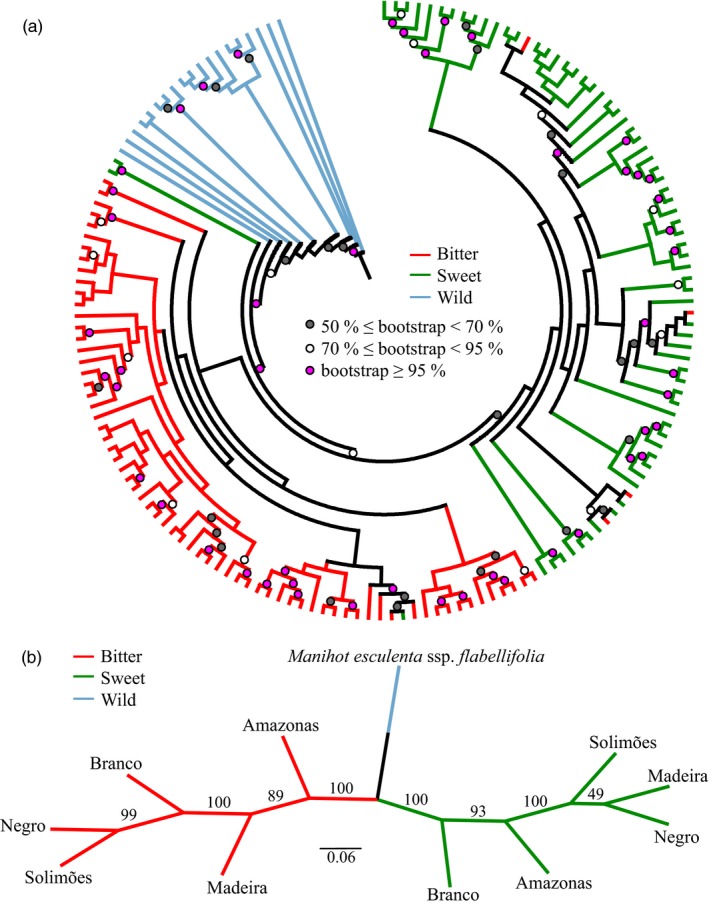
Neighbour‐joining dendrograms showing (a) groupings of individual manioc (*Manihot esculenta*) landraces and (b) groupings of landraces from the major Amazonian rivers. The consensus topologies are based upon 1,000 bootstraps of Cavalli‐Sforza and Edwards (1967) Chord distance estimated from 1,985 neutral SNP markers

The clustering patterns in the individual dendrogram also showed great correspondence with the groups of bitter, sweet and wild manioc (Figure 5a). Wild manioc individuals grouped consistently at the base of the dendrogram. Two consistent groups of bitter and sweet manioc comprised all the cultivated landraces, except for two reputed sweet landraces. We collected these two landraces in the municipality of Oriximá, along the Amazonas River, and they were among the reputed sweet assigned to the bitter cluster in the DAPC. The other reputed bitter landraces that clustered within the sweet group and *vice versa* also exhibited this pattern in DAPC (Figure 3b). The dendrogram for the groups of bitter and sweet manioc from different rivers also suggested the consistency of the bitter–sweet distinction, and wild manioc clustered between these two groups (Figure 5b). This latter dendrogram showed that the relationships among rivers within bitter manioc were distinct from those within sweet manioc, just as in the DAPCs (Figure 4). However, while both analyses recovered similar relationships among rivers within the group of bitter manioc landraces, the relationships were not precisely the same within the group of sweet manioc landraces.

## 4 DISCUSSION

### 4.1 SNPs putatively under selection and the genetic divergence of wild and cultivated manioc

It is not surprising that the outlier SNPs detected in this study had a great variety of putative annotations. Plant domestication involves many different stages from the initial selection to dispersal and adaptation to different agro‐ecological environments (Meyer et al., 2012; Purugganan & Fuller, 2009). Throughout this period, which in the case of manioc is as old as 10,000 BP (Olsen & Schaal, 1999), the crops become better adapted to a great variety of human preferences and agricultural landscapes (Clement, 1999a; Gepts, 2004). Indeed, the elucidation of genes underlining plant domestication and diversification traits showed that these loci have a wide range of functions (Kantar et al., 2017; Meyer & Purugganan, 2013). It is important to stress that we do not aim to associate this set of outlier SNPs to the domestication syndrome in manioc. Because the tests for detecting outlier SNPs used in this study had distinct assumptions, we identified somewhat different sets of markers putatively under selection with each method. Moreover, other approaches, such as genome‐wide association studies (Barrett & Hoekstra, 2011; Ross‐Ibarra, Morrell, & Gaut, 2007), are required to associate genetic polymorphisms to selective advantages and to characterize the functional roles of genes in manioc. It is, however, interesting to note that some outlier SNPs were in transcripts with putative annotations that may have biological meaning in the context of crop domestication and diversification.

Some outlier markers (SNPs 301, 355, 601 and 672) were in predicted genes putatively involved in transcriptional regulation. Frequently, domestication traits are influenced by genes of regulatory factor families (Gepts, 2014; Purugganan & Fuller, 2009). The nodal position of transcription factors within regulatory networks may involve several other genes, and thus, it is more likely that they are involved in adaptations (Lenser & Theißen, 2013). *Zea mays teosinte branched1* (Wang, Stec, Hey, Lukens, & Doebley, 1999) and *Brassica oleracea boCal* (Purugganan, Boyles, & Suddith, 2000) are examples of transcription factors involved in both domestication and diversification. We also identified outlier SNPs in predicted genes putatively encoding proteins with *basic helix‐loop‐helix* (*bHLH*) and *MYB* DNA‐binding domains, which are typical of transcription factors. *Oryza sativa Red pericarp* (*Rc*) (Sweeney, Thomson, Pfeil, & McCouch, 2006) and *Zea mays anthocyanin regulatory C1* (Hanson et al., 1996) are examples of domestication and diversification genes encoding proteins containing these domains.

Outlier SNPs putatively related to moisture (SNP 377) and drought stress (SNP 456), responses to pathogens (SNP 672) and plant defence (SNPs 601 and 1282) make sense in the context of adaptations to distinct ecological environments during domestication and dispersal. Tolerance to biotic and abiotic stresses is essential for crops to adapt to new environments after dispersals and for their deliberate breeding (Allaby et al., 2015; Meyer & Purugganan, 2013). For manioc, such adaptations may have been important during the selection of bitter landraces cultivated in swiddens far from settlements, and thus subjected to natural enemies (Arroyo‐Kalin, 2010), and for the crop's dispersal around the Tropics, since it is well‐adapted to marginal areas (McKey & Delêtre, 2017). Manioc was predicted to be the major crop least sensitive to the predicted effects of climate change in Africa, probably due to its strong abiotic resistance (Jarvis, Ramirez‐Villegas, Campo, & Navarro‐Racines, 2012). Our results suggest that Amazonian manioc landraces may harbour adaptive variation important for the crop's resilience under climate change.

We also found outlier SNPs that may have biological meaning in the context of manioc domestication. Cultivated manioc was selected for larger tuberous roots, but selection also acted on stems (McKey & Delêtre, 2017). It seems that selection for thicker stems of sparsely branched plants to prepare manioc propagules (Elias et al., 2007) modified plant architecture and favoured ease of vegetative propagation. Additionally, the stems of cultivated landraces are more brittle than those of the wild ancestor (McKey & Delêtre, 2017). We found outlier SNPs (SNPs 320 and 1787) putatively involved in root formation and stem development and branching, and putatively related to cell wall firming/softening (SNP 672).

Domestication for vegetative propagation is expected to relax selective pressures on sexual fertility of plants (Zohary, 2004). Although sexual reproduction persists in cultivated manioc (McKey & Delêtre, 2017), flowering ability is quite variable among landraces (Lebot, 2009). In this context, we found three outliers (SNPs 85, 355 and 456) in genes that may have roles in the formation of pollen and flowers. We found that the outlier SNP 1648 was in a gene putatively involved in seed and embryo formation. This SNP may be related to the contrasting seedling morphology of cultivated landraces and wild manioc (Pujol et al., 2005). Wild manioc has seedlings with hypogeal germination (with cotyledons buried in the soil), which guarantees additional opportunities for plant growth in the case of seedling damage. On the other hand, the seedlings of cultivated landraces have epigeal germination (with foliaceous and photosynthetic cotyledons above the soil), which enables fast growth in swiddens (Pujol et al., 2005).

Although our sampling does not represent the extant genetic diversity of *M. esculenta* ssp. *flabellifolia*, we identified many interesting examples of putative adaptive divergence between cultivated landraces and wild manioc. Moreover, the annotation of genes using information from *Arabidopsis* may allow reasonably effective assignment of functionality, even when the organisms are distantly related (Akman, Carlson, Holsinger, & Latimer, 2016). Therefore, these outlier SNPs provide insights into the genomic changes associated with manioc's domestication and diversification, and may serve as preliminary information for future studies aiming at unravelling manioc's domestication genes.

### 4.2 Genome‐wide genetic structure and diversity: Considerations about the evolution of manioc in Amazonia

Although the dendrograms do not clearly support Arroyo‐Kalin’s (2010) hypothesis of an earlier selection of sweet manioc in relation to bitter manioc, all the other results suggest that these groups had independent dispersals. Sweet manioc had a much smaller deficit of heterozygotes than bitter manioc (*f* = 0.048 vs. 0.122, respectively), and the same pattern occurred for these two groups across different rivers. Similar results were reported previously (Alves‐Pereira et al., 2018; Elias et al., 2004; Peroni, Kageyama, & Begossi, 2007), and this trend may reflect a greater diversification period for sweet manioc, as a consequence of an earlier domestication than bitter manioc (Arroyo‐Kalin, 2010). Because bitter manioc cultivation is a major income source for Amazonian smallholders today, a greater deficit of heterozygotes may reflect greater selective pressures for uniformity in comparison with sweet manioc (Alves‐Pereira, Peroni, Abreu, Gribel, & Clement, 2011).

The neutral set of SNP markers revealed contrasting genetic structure and genetic relationships of bitter and sweet manioc across rivers (Figures 4 and 5b). We observed a similar result in our previous study with SSR markers, although the relationships among rivers were not precisely the same (Alves‐Pereira et al., 2018). The SNP markers used in this study suggested a closer relationship between bitter manioc from the Madeira and Amazonas rivers and between sweet manioc from the Amazonas and Branco rivers. Our previous analyses based on SSR markers suggested a great dispersion of both bitter and sweet manioc from the Madeira River across genetic clusters. The bootstrap support of the dendrogram was higher for the analysis based on SNPs than the analysis based on nuclear SSR, but both studies revealed considerable overlap among the landraces from different rivers. Because the current genetic structure of crops may reflect, at least in part, prehistoric events (Miller & Schaal, 2005; Roullier, Rossel, Tay, McKey, & Lebot, 2011), our results based on genome‐wide SNPs strongly suggest separate dispersals for sweet and bitter manioc. Additionally, because divergence among rivers was greater within sweet manioc than within bitter manioc (Table 4, Figure 4), we suggest that the exchange of bitter landraces across Brazilian Amazonia has been more extensive and recent than that of sweet manioc. Exchange networks are common for crops in traditional societies (McKey et al., 2012), and they are frequent for manioc, extending over great geographic distances (Coomes, 2010; Delêtre et al., 2011; Santos, Zárate‐Salazar, Carvalho, & Albuquerque, 2019). Therefore, the exchange networks may increase the opportunities for gene flow between landraces, promoted by the incorporation of volunteer seedlings. However, although contrasting, the levels of genetic divergence among groups of bitter and sweet manioc from different rivers were still low to moderate, suggesting that exchange networks may have a prominent role in shaping the genetic divergence among the major Amazonian rivers (Table 3) and the admixture observed among landraces (Figure 4). Similar trends of low genetic divergence among manioc landraces from different Amazonian regions were recently reported by Sousa, Silva, Dias, Clement, and Sousa (2017). The influence of human‐mediated exchange of manioc landraces certainly comes from pre‐Columbian times, when the major Amazonian rivers were centres of crop diversity (Clement, 1999b). Early exchange networks may have promoted the admixture of landraces that were selected according to distinct preferences. Additionally, many events during the history of human occupation in Amazonia likely contributed to the admixture of manioc landraces (Alves‐Pereira et al., 2018). European colonization decimated Amazonian indigenous populations and certainly caused a great loss of their crops' diversity (Clement, 1999b; Eriksen, 2011). During the 16th century, the surviving native Amazonians moved to more remote areas, and new immigrant populations were established in the next two centuries, especially during the “rubber boom” (1850–1920). These human migrations certainly altered the distribution of Amazonian crop genetic diversity, including manioc and other well‐documented or hypothesized examples, such as Brazil nut (*Bertholletia excelsa*) (Shepard & Ramirez, 2011) and peanuts (*Arachis hypogaea*) (Meggers, 1975).

We found no major patterns of genetic structure along rivers, in accordance with our previous study based on SSR (Alves‐Pereira et al., 2018). However, in the present study the landraces were less dispersed across groups than in the study based on SSR markers, resulting in a somewhat clearer genetic structuring. Therefore, based on the trends of genetic structure inferred with genome‐wide SNPs, we propose some additional hypotheses for the dispersals of bitter and sweet manioc in Amazonia. The greater dispersion of bitter and sweet landraces from the Madeira River in the DAPCs (Figure 4) suggests that this river was important for northwards dispersals of manioc from its centre of domestication in south‐western Amazonia. Bitter manioc along the Solimões River also was well‐dispersed, suggesting that this river may have been a centre of diversity during the early dispersals of bitter manioc, or much later because of its importance during the “rubber boom” (Neves, 2013). The Madeira River was also important for the dispersal of peach palm (*Bactris gasipaes*) from south‐western Amazonia into central and then eastern Amazonia (Clement et al., 2017). Treegourds (*Crescentia cujete*) were probably introduced into Amazonia through the upper Negro and Solimões Rivers (Moreira et al., 2017). Cacao (*Theobroma cacao*) was likely domesticated along the northern Peruvian–Brazilian and southern Colombian–Brazilian borders and was dispersed eastwards into Amazonia along the Solimões and Amazonas Rivers (Thomas et al., 2012). Just as we observed for manioc, these studies revealed complex patterns of genetic structure and highlight how ancient societies managed Amazonian genetic resources (Clement et al., 2015). This is because distinct ethnic preferences in an area of great pre‐Columbian socio‐diversity certainly influenced the selection and dispersals of their crops (Clement et al., 2010; Eriksen, 2011). After millennia of selection, the current high genetic diversity managed by Amazonian smallholders is extremely valuable for the crop's conservation. Bitter and sweet landraces from the Madeira River and bitter landraces from the Solimões River may be used to fill gaps in landrace collections. Additionally, Amazonian landraces may be sources of variation to be used by breeders to purge deleterious mutations of manioc cultivated elsewhere in the world (Ramu et al., 2017).

Given the antiquity of manioc domestication (Olsen & Schaal, 1999) and our limited sampling of ssp. *flabellifolia*, the great divergence between wild and cultivated manioc was expected. However, hybridization between wild and cultivated manioc may still occur (Duputié, David, Debain, & McKey, 2007) and may explain the conspicuous clustering of two sweet landraces from the Amazonas River in the dendrogram (Figure 5a). The dendrogram of individuals revealed a clear separation between the groups of bitter and sweet manioc landraces. This result provided a better resolution of this divergence than our previous analysis based on SSR, in which the sweet landraces formed a less consistent group, suggesting that the bitter–sweet distinction is a primary feature of the crop in Amazonia. Although there was significant genetic divergence between bitter and sweet manioc, we also detected admixed landraces (Figures 3 and 5), which very likely result from the incorporation of volunteer seedlings arising from hybridization between bitter and sweet manioc (Duputié et al., 2009; Martins, 2007). Misassignments of reputed bitter or sweet landraces may be due to genotype x environment interactions (GxE), which has already been reported for cyanogenic levels in manioc (Burns et al., 2012). Nevertheless, the contrasting management practices, such as cultivation in separate swiddens (Martins, 2007) and active selection for variable toxicity levels (McKey & Beckerman, 1993), contribute to explain the persistent genetic differentiation between sweet and bitter manioc. Occasional misassignments may also explain the clustering of the two sweet landraces closer to the group of bitter landraces, given the high bootstrap values supporting this clustering (Figure 5a). Overall, our results suggest that gene flow among bitter and sweet manioc, possible GxE, and hybridization with wild relatives may contribute to maintain the genetic diversity in manioc landraces. Because gene flow among landraces, misassignments and GxE is possible, there will be always some uncertainty in studying the patterns of genetic diversity in pre‐defined groups of bitter and sweet manioc. A clearer picture of the dispersal history of manioc in Amazonia and beyond may emerge with the concomitant determination of cyanogenic potential of the landraces used in the genetic diversity assessment. Although logistically and technically more challenging, such an approach may contribute to minimize the blurring effect caused by the admixture among landraces detected with molecular markers.

Interestingly, the suggestion of GxE and hybridization between Amazonian manioc landraces may have broader implications. For example, in contrast to Amazonia, the toxicity of manioc landraces is a public‐health problem in Africa. Konzo is a health disorder caused by cyanide intoxication commonly reported in manioc‐dependent African communities. Imakumbili, Semu, Semoka, Abass, and Mkamilo (2019) suggested that soil pH and the content of some minerals, such as sulphur and iron, may affect the prevalence of this disorder in Tanzania. Additionally, Bradbury et al. (2013) reported very low genetic divergence between bitter and sweet manioc landraces from African countries, while significant genetic divergence was observed between bitter and sweet manioc landraces from South American countries. It is possible that both hybridization and GxE may influence the accuracy of landrace assignments by African farmers. Therefore, it would be interesting to evaluate the patterns of genetic divergence between sweet and bitter manioc landraces cultivated outside Amazonia to better understand the role of these factors in manioc cultivation. Because the global dispersal of manioc from Amazonia was not necessarily accompanied by cultural appropriation (e.g. original management practices, selection preferences, processing techniques; McKey & Delêtre, 2017), such an analysis would also advance our understanding about the evolutionary dynamics of manioc in different biological and socio‐cultural contexts.

Genetic evidence is essential to advance our knowledge about plant domestication in Amazonia because archaeological and ethnobotanical information is still scarce in this region (Watling et al., 2018). The putative signatures of genomic changes resulting from the domestication and diversification of manioc, and the independent dispersals suggested for bitter and sweet landraces, provide insights into the evolutionary history of the crop. While the complex patterns of neutral genetic structure provide clues about how ancient people managed their native Amazonian crops, the conservation of considerable genetic diversity by current smallholders is essential to maintain the crop's adaptive potential during harsher future climates. Studying the putative signatures of selection and the genetic diversity based on neutral variation of Amazonian crops is fundamental to understand how humans interact with this area of great importance to humanity.

## CONFLICT OF INTEREST

None declared.

## AUTHOR CONTRIBUTIONS

AA‐P, CRC, DP‐R, JBP and MIZ designed the research. AA‐P, CRC, EAV, APS and MIZ got financial support. AA‐P, EAV, GD and SLFR performed data collection. AA‐P, CRC, APS and MIZ performed data analyses and interpretation. AA‐P wrote the manuscript and all authors contributed to its final form.

## Data Availability

Final SNP data uploaded as online Table S1. Sequence alignments (bam files) were deposited in NCBI SRA (accession PRJNA532929).
