# Supplementary material for: A population genomics appraisal suggests independent dispersals for bitter and sweet manioc in Brazilian Amazonia
Source: Evol Appl. 2019 Oct 19;13(2):342–61. doi: 10.1111/eva.12873 (PMC6976959; doi:10.1111/eva.12873)
Supplement: Supplementary file 1 [file EVA-13-342-s001.docx]

**A population genomics appraisal suggests independent dispersals for bitter and sweet manioc in Brazilian Amazonia**

**Supplementary Material – Appendix 1**

**Fig. S1.** Principal component analysis (PCA) performed with *pcadapt* to detect outlier SNPs putatively under selection. The analysis was based on 2,013 SNP markers identified in 159 accessions of *Manihot esculenta* (bitter and sweet landraces and wild individuals) from the major Amazonian rivers in Brazil. A) Scree plot showing the proportion of explained variance in PCA for the first *K* = 20 principal components. The number of components retained in the analysis was *K* = 3, chosen as the point where the curve inflects to the right and presents decreasing explanations (Cattell’s rule) to avoid retaining components that explain random variation. B) Scatter plot of the first two principal components showing the dispersion of individuals. The first principal component explains the divergence between wild and cultivated manioc, while the second principal component explains the divergence between bitter and sweet manioc landraces.

**
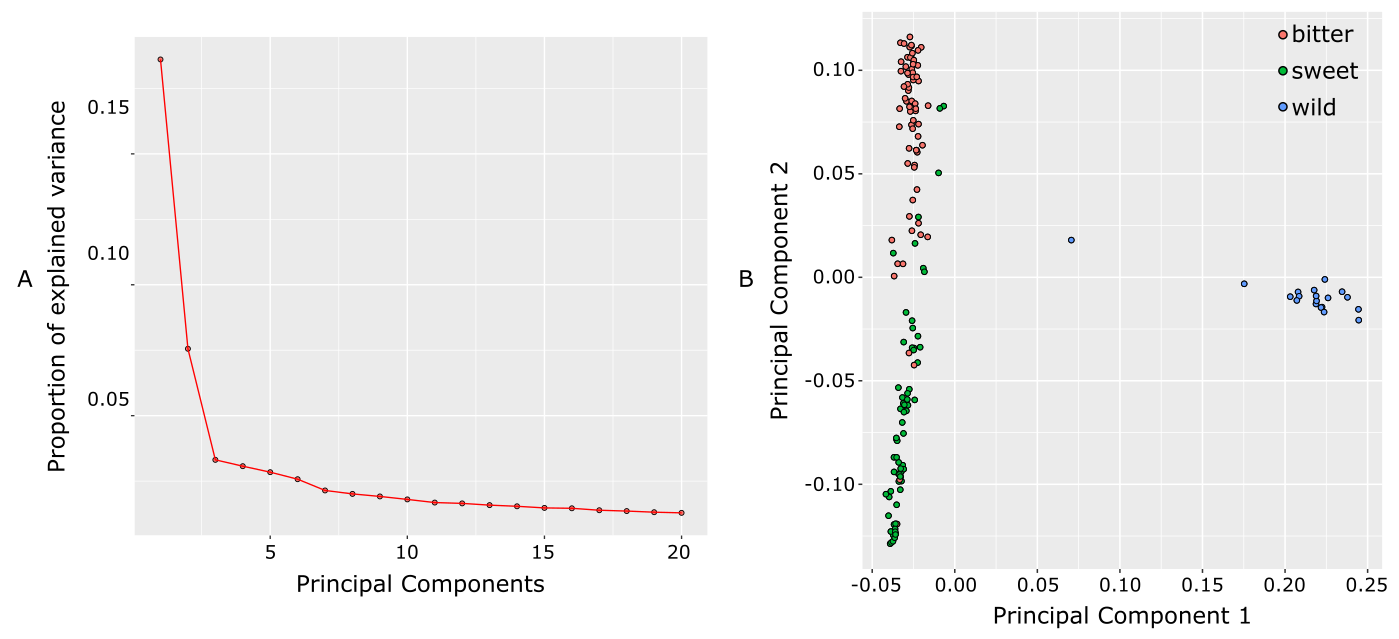
**

**Table S1.** Collection sites and number of manioc (*Manihot esculenta* Crantz) landraces and wild individuals sampled. Municipalities within rivers with their respective numbers of bitter, sweet, and wild manioc sampled. Geographic coordinates indicate the municipality seats.

| **River** | **Location** | **Manioc** | | | |
| --- | --- | --- | --- | --- | --- |
| Municipality |  | **bitter** | **sweet** | **wild** | **Total** |
| **Amazonas** |  | **12** | **18** | **-** | **30** |
| Almeirim | 01°31'22" S 52°34'55" W | - | 3 | - | 3 |
| Monte Alegre | 01°59'56" S 54°04'58" W | 2 | 3 | - | 5 |
| Oriximiná | 01°45'57" S 55°51'57" W | 1 | 5 | - | 6 |
| Parintins | 02°37'40" S 56°44'09" W | 3 | 5 | - | 8 |
| Santarém | 02°26'34" S 54°42'28" W | 6 | 2 | - | 8 |
| **Branco** |  | **7** | **8** | **-** | **15** |
| Bonfim | 03°21'36" N 59°49'58" W | 3 | 3 | - | 6 |
| Caracaraí | 01°48'57" N 61°07'40" W | 4 | 5 | - | 9 |
| **Madeira** |  | **19** | **18** | **-** | **37** |
| Borba | 04°23'16" S 59°35'38" W | 6 | - | - | 6 |
| Guajará Mirim | 10°46'58" S 65°20'22" W | 5 | 4 | - | 9 |
| Humaitá | 07°30'22" S 63°01'15" W | 3 | 3 | - | 6 |
| Manicoré | 05°48'32" S 61°18'00" W | 5 | 5 | - | 10 |
| Novo Aripuanã | 05°08'00" S 60°22'30" W | - | 6 | - | 6 |
| **Negro** |  | **16** | **9** | **-** | **25** |
| Barcelos | 00°58'30" S 62°55'26" W | 4 | 5 | - | 9 |
| Santa Isabel do Rio Negro | 00°24'50" S 65°01'08" W | 4 | 4 | - | 8 |
| São Gabriel da Cachoeira | 00°07'48" S 67°05'20" W | 8 | - | - | 8 |
| **Solimões** |  | **17** | **16** | **-** | **33** |
| Alvarães | 03°13'15" S 64°48'15" W | 5 | 2 | - | 7 |
| Autazes | 03°34'48" S 59°07'51" W | 5 | 4 | - | 9 |
| Codajás | 03°50'13" S 62°03'25" W | 2 | 1 | - | 3 |
| Fonte Boa | 02°30'50" S 66°05'30" W | - | 2 | - | 2 |
| Manaquiri | 03°25'41" S 60°27'34" W | 3 | 4 | - | 7 |
| São Paulo de Olivença | 03°22'40" S 68°52'22" W | - | 2 | - | 2 |
| Santo Antônio do Içá | 03°06'07" S 67°56'24" W | - | 1 | - | 1 |
| Tabatinga | 04°15'09" S 69°56'17" W | 2 | - | - | 2 |
| **Wild manioc** |  | **-** | - | **19** | **19** |
| Guajará Mirim | 10°46'58" S 65°20'22" W | - | - | 1 | 1 |
| Rolim de Moura | 11°48'13" S 61°48'12" W | - | - | 18 | 18 |
| **Total** |  | **71** | **69** | **19** | **159** |

**Appendix 2 (Separate Excel file)**

**Table S1.** Genetic matrix of 2,031 SNP markers identified for 159 accessions of *Manihot esculenta* (bitter and sweet landraces and wild individuals) from the major Amazonian rivers in Brazil. Data is coded in the Variant Call Format (VCF). (Excel file)

**Table S2.** List of SNP markers putatively under selection for each of the three tests (*BayeScan*, *pcadapt* and *fsthet*) performed 159 accessions of *Manihot esculenta* (bitter and sweet landraces and wild individuals) from the major Amazonian rivers in Brazil. (Excel file)

**Table S3.** Annotation of 46 SNP markers putatively under selection based on two of the three tests (*BayeScan*, *pcadapt* and *fsthet*) performed for 159 accessions of *Manihot esculenta* (bitter and sweet landraces and wild individuals) from the major Amazonian rivers in Brazil. (Excel file)

**Table S4**. Membership coefficients from discriminant analysis of principal components (DAPCs) based on 1,985 neutral SNP markers for 159 accessions of *Manihot esculenta* (bitter and sweet landraces and wild individuals) from the major Amazonian rivers in Brazil. These results were used to produce the bar plot of Fig. 3b and the pie charts of Figs 4b, d of the main text. (Excel file)
